# Supplementary material for: Smooth Interpolating Curves with Local Control and Monotone Alternating Curvature
Source: Comput Graph Forum. 2022 Oct 6;41(5):25–38. doi: 10.1111/cgf.14600 (PMC9827861; doi:10.1111/cgf.14600)
Supplement: Supplementary file 1 — Supplement Material [file CGF-41-25-s001.zip › Local-Smooth-Interpolating-MonoCurvature/extern/clothoids/submodules/Utils/docs/api-cpp/program_listing_file_Trace.cc.html]

Program Listing for File Trace.cc — Utils v1.2

### Navigation

- index
- toc
- Utils »
- Program Listing for File Trace.cc

# Program Listing for File Trace.cc¶

↰ Return to documentation for file (`Trace.cc`)

```
/*--------------------------------------------------------------------------*\
 |                                                                          |
 |  Copyright (C) 2017                                                      |
 |                                                                          |
 |         , __                 , __                                        |
 |        /|/  \               /|/  \                                       |
 |         | __/ _   ,_         | __/ _   ,_                                |
 |         |   \|/  /  |  |   | |   \|/  /  |  |   |                        |
 |         |(__/|__/   |_/ \_/|/|(__/|__/   |_/ \_/|/                       |
 |                           /|                   /|                        |
 |                           \|                   \|                        |
 |                                                                          |
 |      Enrico Bertolazzi                                                   |
 |      Dipartimento di Ingegneria Industriale                              |
 |      Universita` degli Studi di Trento                                   |
 |      email: enrico.bertolazzi@unitn.it                                   |
 |                                                                          |
\*--------------------------------------------------------------------------*/

#ifndef DOXYGEN_SHOULD_SKIP_THIS

#include "Utils.hh"

#ifdef UTILS_OS_WINDOWS
#include <windows.h>
#else
#include <execinfo.h> // for backtrace
#include <dlfcn.h>    // for dladdr
#include <cxxabi.h>   // for __cxa_demangle
#include <sys/types.h>
#include <unistd.h>
#endif

#ifdef __clang__
#pragma clang diagnostic ignored "-Wpoison-system-directories"
#pragma clang diagnostic ignored "-Wc++98-compat"
#endif

#ifdef UTILS_OS_OSX
  //#define UNW_LOCAL_ONLY
  #include <libunwind.h>
#endif

namespace Utils {

  using std::runtime_error;
  using std::ostringstream;
  using std::string;
  using std::hex;
  using std::dec;

  const char*
  Runtime_Error::what() const noexcept {
    return runtime_error::what();
  }

  const char*
  Runtime_TraceError::what() const noexcept {
    return runtime_error::what();
  }

  #ifdef UTILS_OS_WINDOWS

  void
  printTrace(
    int                 line,
    char const * const  file,
    string const      & msg,
    ostream_type      & stream
  ) {
    fmt::print( stream,
      "---------------------------------------------------------\n"
      "file: {}:{}\n{}\n"
      "---------------------------------------------------------\n",
      file, line, msg
    );
    #ifndef __MINGW32__
    ULONG const framesToSkip = 0;
    ULONG const framesToCapture = 64;
    void* backTrace[framesToCapture] {};
    ULONG backTraceHash = 0;
    USHORT const nFrame = CaptureStackBackTrace(
      framesToSkip, framesToCapture, backTrace, &backTraceHash
    );
    for ( USHORT iFrame = 0; iFrame < nFrame; ++iFrame )
      fmt::print( stream, "[{}] = {}\n", iFrame, backTrace[iFrame] );
    fmt::print( stream, "backTraceHash = {0:x}\n", backTraceHash );
    #endif
  }

  string
  Runtime_TraceError::grab_backtrace(
    string const &     reason,
    char const * const file,
    int                line
  ) const {
    return fmt::format( "\n{}\nOn File:{}:{}\n", reason, file, line );
  }

  #else

  static
  inline
  string
  demang( char const * const mangled_name ) {
    if ( mangled_name == nullptr ) return string("");
    int status = 0 ;
    string retval = mangled_name;
    char * name = abi::__cxa_demangle( mangled_name, nullptr, nullptr, &status );
    if ( status == 0 ) {
      retval = name;
      // extract only name
      char const * p = strchr(name,'(');
      if ( p != nullptr ) retval = retval.substr(0,p-name);
    }
    if ( name != nullptr ) free(name) ;
    return retval;
  }

  void
  printTrace(
    int                line,
    char const * const file,
    string const     & reason,
    ostream_type     & stream
  ) {

    fmt::print(
      stream, "\n{}\nOn File:{}:{}\nprocess ID:{}, parent process ID:{}\nstack trace:\n",
      reason, basename(file), line, getpid(), getppid()
    );

    #ifdef UTILS_OS_OSX
    unw_cursor_t cursor;
    unw_context_t context;

    // Initialize cursor to current frame for local unwinding.
    unw_getcontext(&context);
    unw_init_local(&cursor, &context);

    // Unwind frames one by one, going up the frame stack.
    while ( unw_step(&cursor) > 0 ) {
      unw_word_t offset, pc;
      unw_get_reg(&cursor, UNW_REG_IP, &pc);
      if ( pc == 0 ) break;
      stream << "0x" << hex << pc << ":" << dec;
      char sym[256];
      if ( unw_get_proc_name(&cursor, sym, sizeof(sym), &offset) == 0 ) {
        stream << " (" << demang( sym ) << "+0x" << hex << offset << ")\n" << dec;
      } else {
        stream << " -- error: unable to obtain symbol name for this frame\n";
      }
    }
    #else
    // record stack trace upto 128 frames
    void *callstack[128] = {};

    // collect stack frames
    int frames = backtrace( callstack, 128 );

    // get the human-readable symbols (mangled)
    char** strs = backtrace_symbols( callstack, frames );

    for ( int i = 1; i < frames; ++i) {
        Dl_info dlinfo;
        if( !dladdr(callstack[i], &dlinfo) ) continue;
        fmt::print( stream, "{:2} {}\n", i, demang( dlinfo.dli_sname ) );
    }
    free(strs);
    #endif
  }

  string
  Runtime_TraceError::grab_backtrace(
    string const &     reason,
    char const * const file,
    int                line
  ) const {
    ostringstream ost;
    printTrace( line, file, reason, ost );
    return ost.str();
  }
  #endif

}

#endif
```

### Quick search

### Table of Contents

- rang - Colors for your Terminal
- Zstr
- C++ API

«
hide menu

menu
sidebar
»

### Navigation

- index
- toc
- Utils »
- Program Listing for File Trace.cc

© Copyright 2021, Enrico Bertolazzi.
Created using Sphinx 4.2.0.
